# Symbiotic Associations in the Phenotypically-Diverse Brown Alga *Saccharina japonica*

**DOI:** 10.1371/journal.pone.0039587

**Published:** 2012-06-20

**Authors:** Evgeniy S. Balakirev, Tatiana N. Krupnova, Francisco J. Ayala

**Affiliations:** 1 Department of Ecology and Evolutionary Biology, University of California Irvine, Irvine, California, United States of America; 2 A. V. Zhirmunsky Institute of Marine Biology, Far Eastern Branch, Russian Academy of Science, Vladivostok, Russia; 3 Pacific Research Fisheries Centre (TINRO-Centre), Vladivostok, Russia; University of Lausanne, Switzerland

## Abstract

The brown alga *Saccharina japonica* (Areschoug) Lane, Mayes, Druehl et Saunders is a highly polymorphic representative of the family Laminariaceae, inhabiting the northwest Pacific region. We have obtained 16S rRNA sequence data in symbiont microorganisms of the typical form (TYP) of *S. japonica* and its common morphological varieties, known as “longipes” (LON) and “shallow-water” (SHA), which show contrasting bathymetric distribution and sharp morphological, life history traits, and ecological differences. Phylogenetic analysis of the 16S rRNA sequences shows that the microbial communities are significantly different in the three forms studied and consist of mosaic sets of common and form-specific bacterial lineages. The divergence in bacterial composition is substantial between the TYP and LON forms in spite of their high genetic similarity. The symbiont distribution in the *S. japonica* forms and in three other laminarialean species is not related to the depth or locality of the algae settlements. Combined with our previous results on symbiont associations in sea urchins and taking into account the highly specific character of bacteria-algae associations, we propose that the TYP and LON forms may represent incipient species passing through initial steps of reproductive isolation. We suggest that phenotype differences between genetically similar forms may be caused by host-symbiont interactions that may be a general feature of evolution in algae and other eukaryote organisms. Bacterial symbionts could serve as sensitive markers to distinguish genetically similar algae forms and also as possible growth-promoting inductors to increase algae productivity.

## Introduction

Polymorphism in morphological traits is widespread in marine organisms. However, the factors that influence this diversity, its evolutionary roots, functional role, and underlying mechanisms remain largely unknown [1]. The problem is partly due to difficulties to maintain and breed marine organisms in laboratory conditions. In the absence of information from genetically defined crosses, it is not possible to determine the extent to which morphological variability is genetically based. A few studies have shown the existence of genetic differentiation between morphological forms. Indirect evidence, based on the correspondence between the spatial distribution of genetic variation and morph frequencies, were obtained for the ascidian *Botryllus schlosseri* [2], crustacean *Idotea balthica* [3], mollusks *Littorina mariae* [4], *Nucella lapillus* [5], and *Argopecten purpuratus* [6], and for sea urchins *Echinometra* [7]. However, in many other cases, there is no clear explanation of morphological polymorphism. Multiple examples of morphological variants with uncertain taxonomical status have been described for marine organisms including algae (e.g., [8]–[12]). Morphological polymorphism may likely reflect phenotypic plasticity driven by diet or other environmental factors.

Here we focus on the brown alga *Saccharina japonica* (Areschoug) Lane, Mayes, Druehl et Saunders (previously *Laminaria japonica*; [13]) inhabiting a wide range of the northwest Pacific region. Morphological forms with uncertain taxonomical status are common in this alga [14]–[15]. The typical form of *S. japonica* (TYP) is 2.0–3.5 m length and it inhabits the littoral zone at preferred depths 5–11 m, with wide geographical distribution. The deep-water (or “longipes”) form of *S. japonica* (LON) may reach 6.0–8.0 m and it inhabits the sublittoral zone at preferred depths 14–25 m. The LON form has a relatively restricted distribution in the Sea of Japan and the Sea of Okhotsk and it grows in compact settlements, separately from the TYP form at a significant distance (300–1000 m) from sea-shore. The TYP and LON forms have substantial differences in morphology, reproductive biology, ecology, and other important features, exhibited from the first year of algal life, which motivated taxonomical consideration of the LON form as a separate species [15]. However, transplant experiments [16]–[17] and genetic data [18] do not corroborate a distinct species status for the LON form. The shallow-water form of *S. japonica* (SHA) inhabits the supralittoral zone (0.1–0.5 m depth) and it is widely distributed in the Primorye coast region, Sea of Japan. (For more detailed characteristics of the *Saccharina* forms see [18].).

The TYP and LON forms are genetically very similar in spite of their drastic differences in morphology, life history traits, and ecology [18]. However, the SHA form is genetically different from the TYP and LON forms and it is closely related to *S. cichorioides*. Thus, there is no consistent relationship between morphological and genetic divergence, suggesting that fertility barriers may arise without affecting genetic divergence (at least in the particular genes investigated). Indeed, genetically similar (or identical) but morphological different variants have been observed in algae [19], [20], sea urchins [21], and other marine organisms (see references above).

Previously [21] we investigated the bacterial symbionts in sea urchin *Strongylocentrotus intermedius* and found that, in spite of their high genetic similarity, the two morphological variants (U and G forms) predominantly harbored highly divergent bacterial lineages belonging to two different taxonomic classes, Flavobacteria and Sphingobacteria. Now we present the data on symbiont microorganisms for the morphological forms of the brown alga *Saccharina japonica*. We investigate the bacterial symbionts of the *S. japonica* morphological forms, because algae bacterial symbionts have diverse and important roles in the nutrition, defense, recognition, and other host functions (review in [22]), which in turn may promote evolutionary changes in the hosts. In spite of intensive investigations of bacterial symbionts in algae [22], there are no data concerning the evolutionary association between the symbioses and particular algae morphological forms. We have analyzed the symbiont composition of the TYP, LON, and SHA forms using 16S rRNA sequences, seeking to ascertain the basis for their drastic morphological differences and to understand the discrepancy between morphology and genetics.

We have found that the TYP, LON, and SHA morphological forms have significantly different symbiont associations. The divergence in bacterial composition is substantial between the TYP and LON forms in spite of their high genetic similarity. Taking into account that symbiotic bacteria can have significant roles in eukaryotic evolution (e.g., [23]–[25]), we propose that the TYP and LON forms of *S. japonica* represent distinct ecomorphological adaptations to contrasting shallow- and deep-water marine environments and might be considered incipient species. We also propose that symbiotic bacteria could be an important causative agent leading to morphological and potentially genetic divergence in the algae studied, and that bacterial symbionts might account for the fertility barriers between genetically similar algae lineages. The data on bacterial symbionts could have practical applications in algae mariculture, serving as sensitive markers for genetically similar forms and as growth promoting agents to improve alga productivity.

## Results

### Phylogenetic Affiliation of the Bacterial 16S rRNA Clones

**Figure 1:**
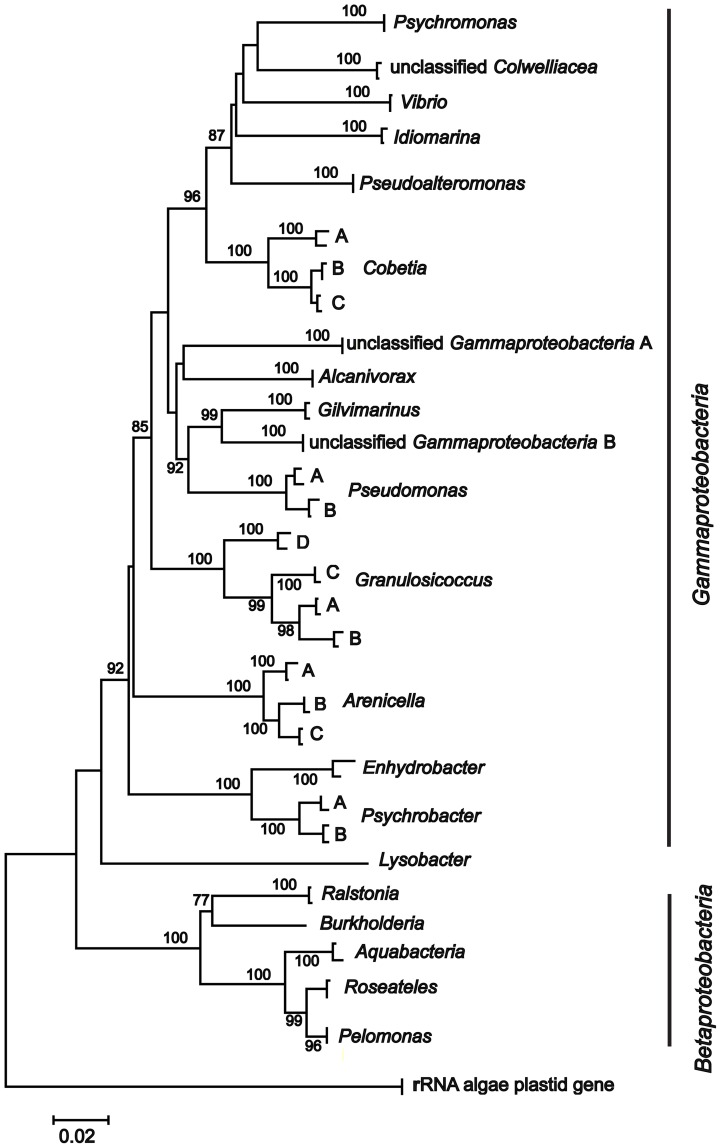
Neighbor-joining tree of 16S rRNA sequences of *Saccharina japonica* symbionts, based on Kimura's 2-parameter distance. We used two representative phylotypes to illustrate bacterial diversity for each *Proteobacteria* genus. The numbers at the nodes are bootstrap percent probability values based on 10,000 replications. rRNA algae plastid genes are used as outgroup. *Gammaproteobacteria* and *Betaproteobacteria* phylotypes are marked by vertical lines. Other comments see Table 1.

We cloned and sequenced the fragments of the bacterial 16S rRNA gene for nine individuals of *S. japonica*, three individuals for each, the TYP, LON, and SHA forms. Additionally we analyzed bacterial symbionts in three laminarialean species, *Alaria marginata*, *Tauya basicrassa*, and *Arthrothamnus bifidus*. The non-chimeric sequences (totally 397 clones) were used for phylogenetic analysis that detected 23 bacterial phylotypes for the TYP, LON, and SHA forms of *S. japonica* and a total of 32 phylotypes including the three other laminarialean species (Table 1). A BLAST search of each clone found close matches with multiple bacteria belonging to the phylum *Proteobacteria* (Table 1). Most of the inferred microorganisms were members of two classes: *Gammaproteobacteria* and *Betaproteobacteria*; 16 additional sequences were associated with the phylum *Cyanobacteria* and represented the algae rRNA mitochondrial and chloroplast sequences probably derived from ancient symbionts (data not shown). Fig. 1 represents the bacterial diversity (only two sequences of each phylotype were used for the tree construction).

**Table 1:** Bacterial symbiont composition in three *Saccharina japonica* forms (TYP, LON, and SHA) and in three other laminarialean algae, *Tauya basicrassa* (TAY), *Arthrothamnus bifidus* (ART), and *Alaria marginata* (ALA).

	TYP (97)	LON (97)	SHA (83)	TAY (54)	ART (34)	ALA (32)
Gammaproteobacteria						
*Psychromonas*	-	7	-	-	-	-
unclassified *Colwelliaceae*	7	-	-	-	-	-
*Vibrio*	-	9	-	-	-	-
*Idiomarina*	5	-	-	-	-	-
*Pseudoalteromonas*	-	38	12	1	7	-
*Cobetia* A	-	3	-	-	-	-
*Cobetia* B	-	27	2	-	1	20
*Cobetia* C	-	-	-	4	-	-
*Alcanivorax*	5	-	-	-	-	-
unclassified *Gammaproteobacteria* A	-	-	3	-	-	-
*Arenicella* A	-	-	7	-	-	-
*Arenicella* B	4	3	-	-	-	-
*Arenicella* C	37	5	4	-	-	-
*Pseudomonas* A	-	-	-	-	2	-
*Pseudomonas* B	-	-	-	12	-	-
*Gilvimarinus*	4	-	-	-	-	-
unclassified *Gammaproteobacteria* B	13	-	-	-	-	-
*Granulosicoccus* A	7	2	40	-	-	-
*Granulosicoccus* B	-	-	3	-	-	-
*Granulosicoccus* C	10	3	2	-	-	-
*Granulosicoccus* D	-	-	2	-	-	-
*Psychrobacter* A	-	-	2	-	-	-
*Psychrobacter* B	-	-	-	18	-	-
unclassified *Alteromonadales*	-	-	2	-	-	-
*Lysobacter*	-	-	-	1	-	-
*Enhydrobacter*	-	-	-	4	-	-
Betaproteobacteria						
*Ralstonia*	5	-	-	3	17	2
*Aquabacterium*	-	-	-	1	2	-
*Roseateles*	-	-	3	1	-	8
*Pelomonas*	-	-	1	8	1	-
*Acidovorax*	-	-	-	-	4	2
*Burkholderia*	-	-	-	1	-	-

For abbreviations, see Material and Methods. Total number of phylotypes (using 97% criterion of similarity) for each of the three *S. japonica* forms and three additional species are shown in parentheses at the top. A, B, C, and D: different phylotypes within a bacterial genus.

The clones belonging to *Gammaproteobacteria* and *Betaproteobacteria* were unequally distributed between the TYP, LON, and SHA forms. These forms were predominantly associated with *Gammaproteobacteria*; the *Betaproteobacteria* phylotypes were rare (paired t-test  = 12.54, *P*<0.001; see Table 1). The deviation from equal proportion of *Gammaproteobacteria* and *Betaproteobacteria* was highly significant separately for the TYP (χ^2^ = 45.33, df = 1, *P*<0.001), LON (χ^2^ = 38.50, df = 1, *P*<0.001), and SHA form (χ^2^ = 42.50, df = 1, *P*<0.001). However, the *Gammaproteobacteria* and *Betaproteobacteria* phylotypes from TAU, ART, and ALA were not distinctly distributed along the trees (χ^2^ = 2.12, df = 1, *P*>0.05).

The *Gammaproteobacteria* phylotypes had close matches with twelve described genera (Table 1). A number of *Gammaproteobacteria* phylotypes formed a pretty divergent cluster of sequences without any close matching from the RDP and Greengenes databases. We denote them as unclassified *Gammaproteobacteria* A and B (Table 1; Fig. 1). The *Betaproteobacteria* clones formed a number of divergent clusters phylogenetically close to bacteria from four described genera (Table 1). Most of the bacteria lineages were previously detected in laminarialean algae [26]–[29]; review in [12].

### *Proteobacteria* Community Composition

Bacterial communities of the TYP, LON, and SHA forms represent a mosaic distribution of common and form-specific bacterial lineages (Fig. 1; Table 1). Common phylotypes present in all three morphological forms include *Granulosicoccus* A and C and *Arenicella* C (3 out of the 23 phylotypes). The only common phylotype for TYP and LON forms includes *Arenicella* B (1 out of 23; hereafter excluding common phylotypes present in all three morphological forms); common phylotypes for LON and SHA forms include *Pseudoalteromonas* and *Cobetia* B (2 out of 23); there are no common phylotypes for TYP and SHA forms. Most common phylotypes were detected for the LON and SHA forms, which have the most divergent bathymetric distribution (see Introduction).

Each morphological form had specific bacterial lineages. TYP specific phylotypes included unclassified *Colwelliaceae*, *Idiomarina*, *Alcanivorax*, *Gilvimarinus*, unclassified *Gammaproteobacteria* B, and *Ralstonia* (6 out of 23); LON specific bacterial lineages included *Psychromonas*, *Vibrio*, and *Cobetia* A (3 out of 23); SHA specific bacterial lineages included unclassified *Gammaproteobacteria* A, *Arenicella* A, *Granulosicoccus* B and D, *Psychrobacter* A, unclassified *Alteromonadales*, *Roseateles*, and *Pelomonas* (8 out of 23) (Table 1).

Frequency distribution of phylotypes was highly non-uniform: each algal form had a number of prevalent (non unique) phylotypes as well as phylotypes represented by single sequences (Table 1). Prevalent and form-specific phylotypes might be considered as diagnostic taxonomical markers that clearly distinguish the TYP, LON, and SHA forms. Unclassified *Gammaproteobacteria* B, unclassified *Colwelliaceae*, *Alcanivorax*, *Idiomarina*, and *Ralstonia* were diagnostic for TYP; *Psychromonas*, *Vibrio*, and *Cobetia* A were diagnostic for LON; unclassified *Gammaproteobacteria* A, *Arenicella* A, *Granulosicoccus* B, and *Roseateles* were diagnostic for SHA (Table 1). Interestingly, the number of “diagnostic” phylotypes was noticeably greater between the genetically similar TYP and LON forms than between the genetically divergent SHA and either the TYP or LON forms. Thus, the absence of genetic differences between the TYP and LON forms is counterbalanced by significant differences in bacterial composition that might serve as a factor for reproductive isolation and morphological divergence between the TYP and LON forms (see below).

**Figure 2:**
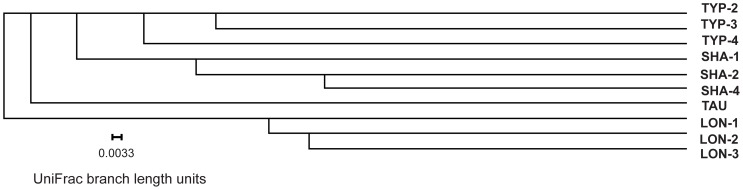
UPGMA distance tree of the *Saccharina japonica* morphological forms based on the bacterial contents they carry. The scale bar shows the distance between clusters in UniFrac units [31]: a distance of 0 means that two environments are identical, and a distance of 1 means that two environments contain mutually exclusive lineages. Scale: 1 dash ∼ 0.0033 branch length units. Three individuals for each morphological form, the TYP (TYP-2, TYP-3, and TYP-4), LON (LON-1, LON-2, and LON-3), and SHA (SHA-1, SHA-2, and SHA-4) are used for the bacterial content analysis. For sample abbreviations see “Materials and Methods”.

Using Martin's [30] and Lozupone and Knight's [31] methods we detected statistically significant differences between the bacterial communities associated with the TYP, LON, and SHA forms of *S. japonica*: *P*<0.01 corrected for multiple comparisons. This result indicates that the sequences are significantly clustered by overall environment. The UniFrac tests were also highly significant for each sample, *P*<0.01 corrected for multiple comparisons, indicating that the *Gammaproteobacteria* and *Betaproteobacteria* sequences obtained from the *S. japonica* TYP, LON, and SHA forms represent a significant incidence of unique branch length. Interestingly, once again, the divergence in bacterial composition is substantial between the TYP and LON forms in spite of their high genetic similarity. All distances obtained with the UniFrac metric (Fig. 2) are fairly similar (ranging from 0.64 to 0.77) and not correlated with the genetic relationships between the forms. The highest distance (0.7722) occurs between the LON and TYP forms that exhibit no genetic differences [18]. The distances between the SHA and the other two forms are lower (0.6864 between TYP and SHA; 0.6347 between LON and SHA) though in this case the two forms are genetically significantly different.

**Figure 3:**
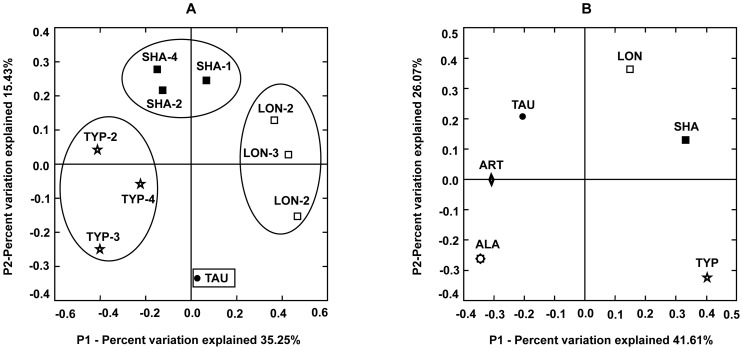
Two principal coordinates from a principal coordinate analysis of the *Proteobacteria* sequences obtained from three morphological forms (A) or species along with the forms (B). The numbers on the axes refer to the percent of the variation explained by each principal component (P1 and P2). Other comments see Figure 2.

Using the raw UniFrac values (data not shown) for all pairs of environments we obtained scatter plots (Fig. 3) of the first two principal coordinates; the different “environments” were represented by the TYP, LON, and SHA *S. japonica* forms (separately for each investigated sample within the form) plus TAU as an outgroup (Fig. 3A) as well as by the *S. japonica* forms (summarized data for each form) and other laminarialean algae, TAU, ART, and ALA (Fig. 3B). The principal components produce biologically meaningful groupings. The two principal components, PC1 and PC2, jointly explain more than half of the variation in the data and separate the TYP–associated bacterial communities from the LON–associated, and SHA–associated communities (Fig. 3A). Importantly, individuals of each morphological form cluster together, supporting the taxonomical validity of the bacterial composition (Fig. 3A). The TAU, ART, and ALA species are dispersed among the *S. japonica* forms; they were collected from different depths (Fig. 3B).

The analysis reveals that the TYP, LON, and SHA morphological forms of the *S. japonica*, sampled from different depths, harbor significantly different bacterial communities. The bacterial profiles suggest that the *S. japonica* morphological forms are not identical with respect to symbiont contents. The bacterial sequences from the other laminarialean species, *Tauya basicrassa*, *Arthrothamnus bifidus*, and *Alaria marginata* are intermingled among the sequences from *S. japonica*, independently of the depth at which they were collected, indicating that the depth of the *S. japonica* fields by itself does not play a determinant role in structuring the bacterial communities.

## Discussion

Previously we found that the TYP and LON morphological forms are genetically very similar [18] and, therefore, they might not be thought of as distinct biological species, as it had been suggested by Gusarova and Ivanova [15]. The SHA form is genetically different from the TYP and LON forms (2.6% total DNA divergence) and it is quite similar (but not identical) to *S. cichorioides*. Thus, the genetic data suggest the existence of two close *Saccharina* lineages inhabiting the Primorye coastal region in the Sea of Japan, *S. japonica* (TYP and LON forms) and *S. cichorioides* (SHA form) [18].

We now found that all three morphological forms, TYP, LON, and SHA have significantly different symbiont associations. The difference between the SHA form versus the TYP plus LON forms is not surprising: these two lineages are genetically different, which may account for the difference in symbiotic compositions. The symbiont differences between TYP and LON forms are less expected, because the forms are identical for all three gene regions studied by Balakirev et al. [18]. Thus, substantial morphological and ecological differences between the TYP and LON forms are consistent with significant differences in their symbiont content but contradict their high genetic similarity.

The present data are similar to our previous results investigating two morphological forms of the sea urchin *Strongylocentrotus intermedius* [21]. As in the case of the *Saccharina* TYP and LON forms, we did not find genetic differences between two sea urchin morphological forms, usual (U) and grey (G), which however had different morphologies and ecological preferences [21]. The low level of genetic divergence between the sea urchin morphological forms sharply contrasted with their bacterial content. The U and G forms were preferentially infected by different bacteria species belonging to two different classes, *Flavobacteria* and *Sphingobacteria*, respectively. Moreover, the pattern of bacterial distribution was very similar in three distantly located geographical settlements of *S. japonica*. The distribution of the symbiotic bacteria in the U and G forms of *S. intermedius* was not related to the depth of settlements [21].

The data obtained for sea urchins and algae suggest that symbiont-induced life history changes may have promoted environmental specialization (shallow- and deep-water preferences for the morphological forms) and might potentially promote speciation in these organisms. Symbiotic bacteria could be an important causative factor leading to morphological and potentially genetic divergence in algae and sea urchins, and it might be a general feature of evolution in other eukaryotes. Taking into account that the opportunity for interbreeding between the forms is lacking, and also the fact that some symbiotic bacteria can have drastic effects on algal morphogenesis and growth [32]–[35], (review in [22]); and also the significant roles of symbiosis in eukaryotic evolution generally (e.g., [23]–[25], [36], [37]), we propose that the TYP and LON forms of *S. japonica* represent distinct eco-morphological adaptations to contrasting littoral- and sublittoral marine environments and might be considered as incipient species, even though their divergence may have occurred recently. We advanced the same suggestion for the sea urchin U and G morphological forms [21].

One possible alternative interpretation of our results is that the difference between the symbionts associated with the morphological forms may be conditioned by the different depths at which the forms settle; that is, it might be the case that different symbionts prevail at different depths. If the settlement depth would be principal factor differentiating the microbial communities we should expect similar symbiont communities for different species collected from the same (or close) depths and more different communities in hosts collected from different depths. This prediction is not supported by the data obtained for the *Bacteroidetes* symbionts of the sea urchin *Strongylocentrotus nudus*: shallow-water *S. nudus* samples have intermingled bacterial distributions belonging to different *Bacteroidetes* lineages, without clear-cut differentiation among the *Bacteroidetes* [21]. Our present data are also inconsistent with the hypothesis: (1) the SHA form has closer bacterial composition to the LON form than to the TYP form, even though LON and SHA come from more contrasting depths (0.5 and 15 m); (2) *Tauya basicrassa*, *Arthrothamnus bifidus*, and *Alaria marginata* have intermingled bacterial distributions belonging to different *Proteobacteria* lineages, without clear-cut differentiation associated with depth. The distribution of the symbiotic bacteria in *Saccharina* and *Strongylocentrotus* is not related to the depth of the settlements; rather, it reflects their phylogenetic position.

The alternative interpretation also contradicts the expectation that bacterial communities in algae and sea urchins are highly specific [21], [38]–[41]; review in [22]. For instance, Ashen and Goff [38] investigated the symbiotic associations between three species of red algae *Prionitis* and gall symbiotic bacteria. They investigated the specificity of these associations by using cross-inoculation trials and found that bacterial symbionts were incapable of inducing galls on alternate hosts [38]. Another example of species specificity of alga-bacterial association, described by Lachnit et al. [41], showed that bacterial communities differ less between regions than between host species, and were more similar in closely related host species. Different species of marine algae growing under close environmental conditions bear different bacterial communities. The host-specific endosymbionts were also detected in the green alga *Caulerpa taxifolia* [39]–[40]. Summarizing the results on chemical interactions between marine macroalgae and bacteria, Goecke et al. [22] conclude that there is a highly specific association of bacterial communities with marine algae. Strict species-specific symbiont-host associations were revealed for other marine organisms (review in [42]). Thus, the specific character of symbiotic bacteria associations as well as the comparative data obtained for species collected from different regions and depths, suggest that the difference in bacterial composition between the algal morphological forms might not simply reflect the different habitats and could play an important role in the morphological and potentially genetic divergence of the alga *S. japonica*, as well as in the sea urchin *S. intermedius*.

Breeding experiments would not be informative for accepting or rejecting alternative hypotheses, because even distantly related algae and sea urchin species produce highly viable first generation hybrids [20], [43]–[48]. The biological species concept in kelps and sea urchins is not particularly practical [19], [20], [46]. For instance, Kraan and Guiry [20] showed that the interspecific DNA sequence divergence in *Alaria* was smaller than the intraspecific sequence divergence. The existence of greater genetic variation within a species than between two species of the same genus casts doubts on the morphological and biological species concepts employed in *Alaria* and on the usefulness of hybridization studies in assessing species-level differences [49]–[52]. Discrepancy between speciation based on morphological versus molecular characteristics results from the different rates at which molecular and morphological changes accumulate [52]. The morphological and biological species concept is not satisfactory for separating sea urchin or algal species, because it does not fully reflect their phylogenetic relationships. Transplant experiments are not decisive either for determining the taxonomical status of the *Saccharina* morphological forms since both the LON form, which is genetically similar to the TYP form, and the SHA form, which is highly different, when being transplanted to the TYP habitat area exhibit drastic morphological transformations that make them morphology indistinguishable from the TYP form [16], [17].

Druehl and Saunders [19] and Kraan and Guiry [20] have shown in the brown alga *Alaria*, that fertility barriers indicating complete reproductive isolation, may arise without any genetic divergence in the particular genes under investigation (review in [53]). However the differences in symbiotic bacteria are consistent with morphological differences. It might be that bacterial symbionts are responsible for the fertility barriers between genetically similar lineages of algae or sea urchins. One possible mechanism may involve pheromone specificity in different morphological forms with different symbiont compositions that might lead to different attraction of spermatozoids by eggs. Sexual reproduction in many algae [54], [55] and sea urchins [56]–[58] involves pheromones which induce behavioral changes in male gametes (sperm chemotaxis). Also adult sea urchin pheromones may attract larvae, which tend to settle near conspecific adults and bacterial biofilm-derived settlement cues from algae induce appropriate settlement sites [59]–[62], as it has also been observed for larvae of many other marine invertebrates [63]. Algae spore settlement and germination has been shown to be determined by bacterial biofilms. The most pronounced stimulating effect for spore settlement has been found in a number of bacteria from the phyla *Proteobacteria* and *Bacteroidetes* (review in [22]). These observations support the hypothesis that bacterial symbionts might be responsible for fertility barriers between genetically similar lineages.

This hypothesis is supported by the highly specific interactions between symbiotic bacteria and algae that are known to influence algae reproductive biology. For instance, Weinberger et al. [64] have shown that spore release in the red alga *Acrochaetium* sp. is bacterially controlled. Also, bacterial symbionts play a role in spore settlement and subsequent colonization of new substrates by algae [65], [66]. Patel et al. [65] detected strict specificity in the settlement-modifying response of bacterial biofilms towards zoospores of marine algae that suggest a bacterial role for the specificity of fertilization in algae. Preferential settlements of spores on specific bacterial biofilms have been observed [65], [67], [68]. A positive correlation between zoospore settlement and bacterial biofilm density points out the important role of bacterial biofilms in the development of algal communities [35]. Ma et al. [69] have recently shown that a significant number of bacterial strains are inhibitory against algal spores, suggesting that bacterial biofilms play an important role in algal spore germination and the subsequent colonization. Symbiotic bacteria could, indeed, be an important causative factor leading to morphological and potentially genetic divergence in marine organisms, including algae and sea urchins.

The bacteria-derived cues from representatives of *Proteobacteria* and *Bacteroidetes* are important in the reproduction and settlement of marine organisms. Moreover, these bacteria are associated with diverse host reproductive manipulations, including cytoplasmic incompatibility (CI), parthenogenesis, and feminization, alterations that may play important roles in the host speciation process [23], [70]–[73]. The potential for CI in contributing to speciation has been extensively discussed (e.g., [74]–[77]). Symbiont-associated changes in dispersal and mating are likely to play a key role in the initiation of genetic differentiation of populations with different infections, because CI can have direct consequences on gene flow between populations, making it a potentially important speciation agent [78]; see additional discussion in [21]. Taking into account the multiple roles of *Proteobacteria* and *Bacteroidetes* in algal and sea urchins reproduction biology, and also considering the fact that morphological forms harbor specific and significantly different symbionts, we suggest that there is a causal connection between symbiont content and the multiple differences observed between algae and between sea urchin forms, which consequently might be considered as incipient species.

In accordance with theoretical inferences, the symbiont-driven evolution could be very fast after the introduction of a new symbiont providing new functional capabilities to a host population. For instance, mating preference (as an early event in speciation) was achieved after only one generation and maintained for at least 37 generations in laboratory populations of *Drosophila melanogaster* reared on different media (molasses and starch medium) [37]. The fly commensal bacteria are responsible for mating preference in *D. melanogaster* by changing the levels of cuticular hydrocarbon sex pheromones [37]. Jiggins and Hurst [79] provide other examples of rapid evolution by symbiont transfer and see symbiont transfers as a sort of “macromutations” that may have a higher selective advantage than “normal” mutations. The morphologically different forms of algae may also be seen as a kind of “macromutations” that open up new avenues for algal diversification.

The data in the present work might have practical applications for *Saccharina* mariculture. For instance, specific bacterial contents detected in different morphological forms could be used as markers for desirable traits. Bacteria associations with humans can serve as a sensitive marker to distinguish human ethnic groups [80] and to infer the migration patterns of human populations [81], providing greater resolution than the analysis of human genes. Another possible practical application is to use the bacterial symbionts for increasing algal biomass production. Traditional artificial selection programs for cultivation of algae, and particularly *Saccharina*, have low efficiency due to low coefficients of heritability for the most important traits (e.g., [82]). Bacterial symbionts (especially with growth promoting effect) may provide a better alternative for agriculture practice (e.g., [83]–[85]). Bacteria from the genus *Halomonas* are capable of improving the growth of the green alga *Dunaliella balwardii* [86], [87]. We observed that a representative of the genus *Cobetia* (belonging to the genus *Halomonas*, family *Halomonadaceae*) is a specific symbiont of the LON form that was associated with extraordinarily strong thallus development and, thus, these bacteria have a potential for enhancing algal growth. We suggest that a bacteria-based approach could be promising for algal cultivation. The use of algae-associated bacteria with growth-promoting abilities can become a mean for maximizing the efficiency of algal cultivation. This approach could significantly improve the traditional selection programs of *Saccharina* and could be an alternative to approaches that use genetically modified (transgenic) organisms (which are still pending for safety approval [88], [89]). Although data on growth promoting bacteria have accumulated significantly, little advantage has been taken to date for artificial selection purposes.

Summarizing our results, we conclude that the bacterial composition of three *S. japonica* morphological forms, TYP, LON, and SHA is significantly different and represents a mosaic distribution of common and form-specific bacterial lineages. There is no consistent relationship between genetic similarity and symbiont composition: genetically similar forms (TYP and LON) are different in symbiont content to the same extent as genetically divergent forms (TYP plus LON vs. SHA). The symbiont distribution in *S. japonica* forms and in three other laminarialean species is not related to the depth of algal settlements; rather it reflects their phylogenetic position. Taking into account the highly specific character of associations between bacteria and algae and that some bacteria can induce drastic morphological changes, including enhancement of algal growth, we propose that the *S. japonica* TYP and LON forms represent distinct ecomorphological adaptations to contrasting marine environments and might be considered incipient species. The results now obtained importantly establish that processes previously observed in sea urchins [21] may not be unique, but rather occur in other very different marine organisms, as now discovered in algae. We also point out that form-specific prevalent bacterial lineages may represent valuable markers for algal taxonomy and symbiont-based algal biomass production.

## Materials and Methods

### Algal Samples

The specimens of S. *japonica* were collected from the Primorye coastal region, Sea of Japan. The TYP and SHA forms were collected near the Cape Dal'niy at depths of 6.0 m and 0.5 m, respectively. The LON form was collected near the Cape Zolotoi at depths of 15.0 m. Additionally we analyzed three laminarialean species from the Okhotsk Sea (a single sample per species): *Alaria marginata* (ALA) (the Odyan Bay, depth of 1.0 m), *Tauya basicrassa* (TAU) (the Tauyskaya Gulf near the island Nedorazumenia, depth of 8.0 m), and *Arthrothamnus bifidus* (ART) (the Babushkin Bay, depth of 1.0 m). No specific permits were required for the described field studies because they did not involve endangered or protected species. The locations are not privately-owned or protected.

### DNA Amplification, Cloning, and Sequences

Total gemonic DNA was extracted using the DNeasy Plant MiniKit protocol (Qiagen, Hilden, Germany) from the meristem part of algae (at the base of the phyloid). This algal part was selected because it was shown previously [27] for *Saccharina latissima* (that is close to *Saccharina japonica*) that in the meristem (along with cauloid) the bacterial communities from different individuals sampled were most stable and specific in different seasons (winter and spring) and geographical location of the sample origin (Baltic Sea and North Sea).

The procedures for DNA amplification, cloning, and sequencing have been described [21], [90]. A 1.5-kb fragment of the 16S rRNA bacterial genes was amplified with primers 5′-tgatcmtggctcagat-3′ (forward) and 5′-taccttgttacgactt-3′ (reverse); these new designed primers avoid abundant co-amplification of the algae chloroplast and mitochondrial rRNA genes, a problem revealed previously in *Laminaria saccharina* [27] (see Text S1 for details). The PCR reactions were carried out in final volumes of 25 µl using TaKaRa Ex Taq™ in accordance with the manufacturer's description (Takara Biotechnology Co., Ltd.). The PCR reaction mixtures were placed in a DNA thermal cycler (Eppendorf, Mastercycler Gradient), incubated 5 min at 95° and subjected to 32 cycles of denaturation, annealing, and extension: 94° for 30 sec, 52° for 30 sec, and 72° for 1.5 min, with a final 7-min extension period at 72°. The PCR products for the 16S rRNA gene were cloned (TOPO TA cloning kit, Invitrogen, Calif.) and sequenced by the dideoxy chain-termination technique using Dye Terminator chemistry and separated with the ABI PRISM 377 automated DNA sequencer (Perkin Elmer). The sequences of both strands were determined for each clone, using overlapping internal primers spaced, on average, 500 nucleotides. At least two independent PCR amplifications were sequenced in both directions to correct for possible cloning or sequencing errors. The sequences were assembled using the program SeqMan (Lasergene, DNASTAR, Inc.). Multiple alignment was carried out manually and using the program CLUSTAL W [91]. The 16S rRNA sequences have been deposited in GenBank under accession numbers JQ218513-JQ218924. The rarefaction curves produced by FastGroupII [92] were monitored to ensure that sufficient numbers of clones were sequenced for each clone library (see Figure S1). The strategy we follow to detect reasonable microbiom representatives is described in more detail in the Text S1. Putative chimeras were identified with the program Bellerophon [93]. We used two programs available in the web, RDP Classifier [94] and Greengenes [95] in order to uncover the bacterial affinities of the *Proteobacteria* clones. We used Martin's [30] and Lozupone and Knight's [31] methods to investigate the structure of the bacterial communities associated with the laminarialean algae, considering each algal individual as an “environment” inhabited by a specific array of bacterial symbionts. The UniFrac test measures the phylogenetic distance between sets of taxa in a phylogenetic tree as the fraction of the branch length of the tree that leads to descendants from either one environment or the other, but not both [31]. The P test uses parsimony to determine whether the distribution of sequences in different environments reflects a history of fewer changes between environments than would be expected by chance [30]. Both, the P test and the UniFrac test can be used to determine whether the communities differ significantly by using Monte Carlo simulations. We measured the overall difference between each pair of morphological forms using the UniFrac metric to assess how far apart the forms are in terms of the microorganisms they share.

## Supporting Information

Figure S1 **Rarefaction analysis of 16S rRNA gene sequences from the three morphological forms of** ***Saccharina japonica*** **: TYP, LON, and SHA.** The total number of sequences (97 for TYP, 97 for LON, and 83 for SHA) is plotted against unique phylotypes defined by using a distance level of 3% calculated by FastGroupII [92]. For the LON and SHA forms four (L24, SPLON29, LON07S7, and LO710) and three (SH1SP3r, ncSH4SP6, and ncSH4SP7) clones, respectively were removed from the analysis because the clones were obtained with the “universal” primers at the preliminary step of work (see Text S1 for details).
(TIF)

Text S1 **Sequencing strategy.**
(DOC)
