# Supplementary figures and images for: Symbiotic Associations in the Phenotypically-Diverse Brown Alga Saccharina japonica
Source: PLoS One. 2012 Jun 20;7(6):e39587. doi: 10.1371/journal.pone.0039587 (PMC3379999; doi:10.1371/journal.pone.0039587)

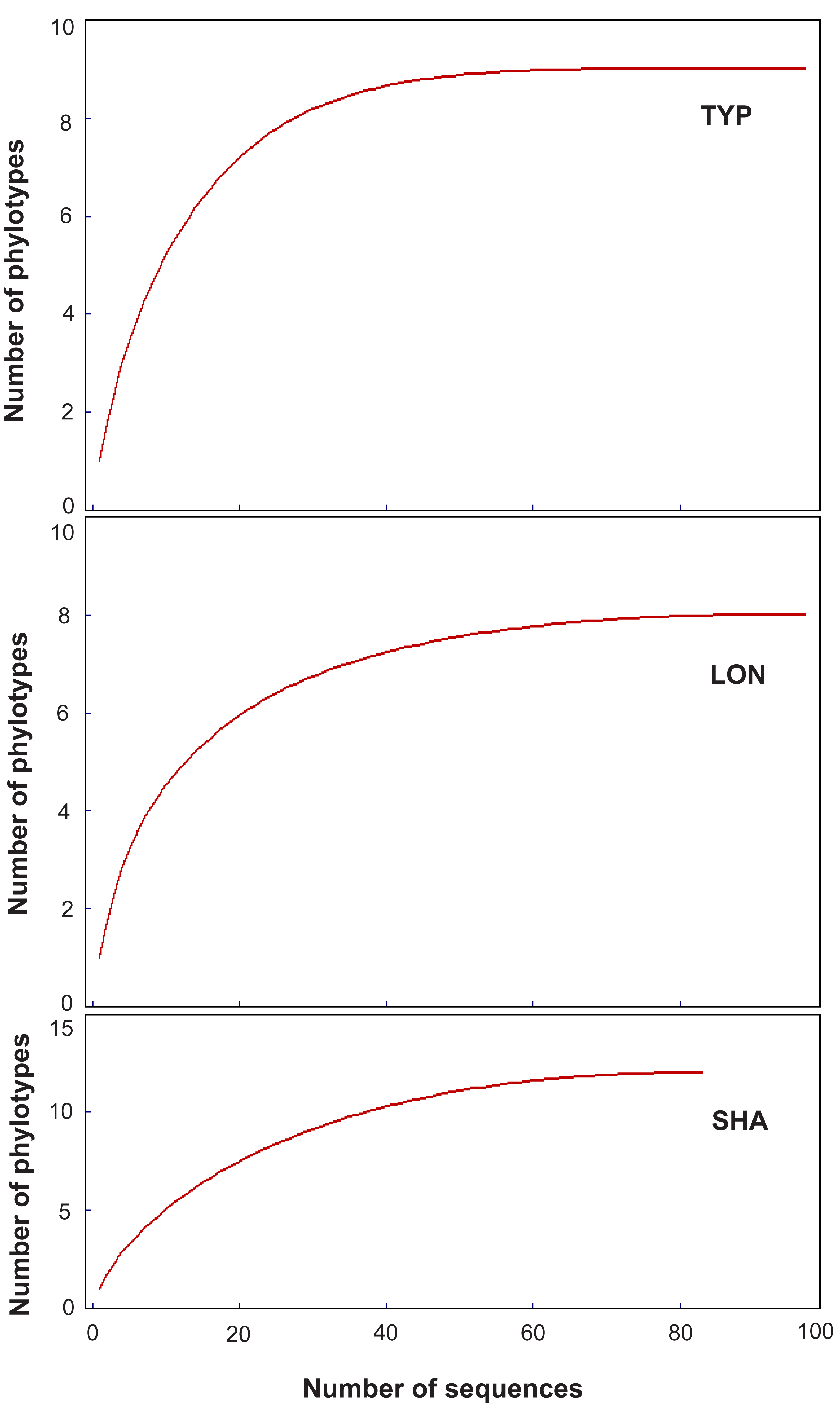

Supplement: Figure S1 — Rarefaction analysis of 16S rRNA gene sequences from the three morphological forms of Saccharina japonica : TYP, LON, and SHA. The total number of sequences (97 for TYP, 97 for LON, and 83 for SHA) is plotted against unique phylotypes defined by using a distance level of 3% calculated by FastGroupII [92]. For the LON and SHA forms four (L24, SPLON29, LON07S7, and LO710) and three (SH1SP3r, ncSH4SP6, and ncSH4SP7) clones, respectively were removed from the analysis because the clones were obtained with the “universal” primers at the preliminary step of work (see Text S1 for details). (TIF) [file pone.0039587.s001.tif]
