# Supplementary material for: Symbiotic Associations in the Phenotypically-Diverse Brown Alga Saccharina japonica
Source: PLoS One. 2012 Jun 20;7(6):e39587. doi: 10.1371/journal.pone.0039587 (PMC3379999; doi:10.1371/journal.pone.0039587)
Supplement: Text S1 — Sequencing strategy. (DOC) [file pone.0039587.s002.doc]

**Supporting information online, Text S1.** Sequencing strategy.

The “universal” bacterial primers (27F and 1492R; Lane, 1991) co-amplify the chloroplast and mitochondrial genes from algae along with the bacterial genes. Staufenberger et al. (2008) revealed 25 mitochondrial and 228 chloroplast algae genes among 333 sequences obtained from the brown alga *Saccharina latissima* (*Laminaria saccharina*) – close brown algae species to *Saccharina japonica* investigated in a present work. Thus, using “universal” primers, around 76% of obtained sequences were not informative. Early in our work we used the “universal” bacterial primers and encountered the same problem of co-amplification of bacterial genes with mitochondrial and chloroplast algal genes. To overcome the problem we designed a new forward primer that prevented co-amplification of algae and bacterial genes (in favor the latter). It was achieved by adding two nucleotides (“AT”) at the 3’ side of the “universal” 27F primer (in bold face font below). The chloroplast and mitochondrial genes from algae have “GA” at positions 21 and 22 but our 16SFSP primer has “AT” at these positions, which prevents co-amplification of the chloroplast and mitochondrial genes from algae and leave the possibility for amplification of genes belonging to bacterial symbionts inhabiting algae only.

-------------------------------------------------------------------------------------------------

1111111111222

1234567890123456789012

“Universal” 27F: 5’-AGAGTTTGATCCTGGCTCAG -3’

Our primer 16SFSP: 5’-TGATCMTGGCTCAG**at**-3’

Mitoch., chloropl. &

Bacteroidetes genes 5’-TGATCMTGGCTCAG**ga**-3’

“Universal” 1492R: 5’-ACGGCTACCTTGTTACGACTT-3’

Our primer 16SRSP: 5’-TACCTTGTTACGACTT-3’

-------------------------------------------------------------------------------------------------

The extent of the phylogenetic cover that could be amplified with our primers is restricted. Particularly we could not amplify the bacteria belonging to the phylum Bacteroidetes because these bacteria also have “GA” at positions 21 and 22 (the conclusion was obtained based on massive alignment of the Bacteroidetes sequences from GenBank; data not shown). However, these bacteria are not frequent in algae. For instance, Staufenberger et al. (2008) investigated bacterial communities associated with brown alga *Saccharina latissima* (*Laminaria saccharina*) – a species that is close to *Saccharina japonica* studied in our work. The algae-associated 16S rRNA gene sequences in *Saccharina latissima* were affiliated to Alpha-, Beta-, and Gammaproteobacteria, as well as to Bacteroidetes (Staufenberger et al. 2008). Among 333 obtained clones Staufenberger et al. (2008) detected only four sequences belonging to Bacteroidetes. Wang et al. (2008) investigated epiphytic marine bacteria of *Saccharina japonica* and revealed mostly Gammaproteobacteria (41 sequences out of 42 obtained). In *Saccharina japonica* we detected bacteria belonging to Beta- and Gammaproteobacteria that are common in laminarialean algae. Thus, our new designed primers avoid contaminant co-amplification of algae chloroplast and mitochondrial genes but still have enough phylogenetic coverage. We also make the primers shorter (16 bp instead of 20-21 bp; see above) to expand the scope of detectable bacterial phylotypes (see Isenbarger et al. 2007).

**References for the Text S1**

1. Beleneva IA, Zhukova NV (2006) Bacterial communities of some brown and red algae from Peter the Great bay, the Sea of Japan. Microbiology 75: 348-357.

2. Burke C, Thomas T, Lewis M, Steinberg P, Kjelleberg S (2011) Composition, uniqueness and variability of the epiphytic bacterial community of the green alga *Ulva australis*. ISME J 5: 590-600.

3. Doolittle WF, Zhaxybayeva O (2009) On the origin of prokaryotic species. Genome Res 19: 744-756.

4. Fraser C, Alm EJ, Polz MF, Spratt BG, Hanage WP (2009) The bacterial species challenge: making sense of genetic and ecological diversity. Science 323:741-746.

5. Isenbarger TA, Finney M, Ríos-Velázquez C, Handelsman J, Ruvkun G (2008) Miniprimer PCR, a new lens for viewing the microbial world. Appl Environ Microbiol 74: 840-849.

6. Lane DJ (1991) 16S/23S rRNA sequencing. P. 115-175. In: Stackebrandt E, Goodfellow M (eds.), Nucleic acid techniques in bacterial systematics. John Wiley & Sons, Chichester, United Kingdom.

7. Lee YK, Jung HJ, Lee HK (2006) Marine bacteria associated with the Korean brown alga, *Undaria pinnatifida*. J Microbiol 44: 694-698.

8. Staufenberger T, Thiel V, Wiese J, Imhoff JF (2008) Phylogenetic analysis of bacteria associated with *Laminaria saccharina*. FEMS Microbiol Ecol 64: 65-77.

9. Uchida M, Maeda T, Shiba T (2002) Phylogenetic analysis of three marine bacteria that have an ability to decompose *Laminaria japonica*. Fish Sci 68: 703-705.

10. Wang G, Shuai L, Li Y, Lin W, Zhao X, et al. (2008) Phylogenetic analysis of epiphytic marine bacteria on Hole-Rotten diseased sporophytes of *Laminaria japonica*. J Appl Phycol 20: 403-409.

11. Wang Z, Xiao T, Pang S, Liu M, Yue H (2009) Isolation and identification of bacteria associated with the surfaces of several algal species. Chinese J Oceanol Limnol 27: 487-492.

12. Wiese J, Thiel V, Nagel K, Staufenberger T, Imhoff JF (2009) Diversity of antibiotic-active bacteria associated with the brown alga *Laminaria saccharina* from the Baltic Sea. Mar Biotechnol 11: 287-300.

13. Yu Y, Breitbart M, McNairnie P, Rohwer F (2006) FastGroupII a web-based bioinformatics platform for analyses of large 16S rDNA libraries. BMC Bioinformatics 7: 57.
